# Can Structural Grading of Foveal Hypoplasia Predict Future Vision in Infantile Nystagmus? A Longitudinal Study

**DOI:** 10.1016/j.ophtha.2019.10.037

**Published:** 2020-04

**Authors:** Sohaib R. Rufai, Mervyn G. Thomas, Ravi Purohit, Catey Bunce, Helena Lee, Frank A. Proudlock, Irene Gottlob

**Affiliations:** 1 The University of Leicester Ulverscroft Eye Unit, Leicester Royal Infirmary, Leicester, United Kingdom; 2 Clinical and Experimental Sciences, Faculty of Medicine, University of Southampton, Southampton, United Kingdom; 3 Department of Primary Care & Public Health Sciences, King’s College London, London, United Kingdom

**Keywords:** FDI, foveal developmental index, IQR, interquartile range, logMAR, logarithm of the minimum angle of resolution, ONL, outer nuclear layer, OS, outer segment, PL, preferential looking, SD, standard deviation, VA, visual acuity

## Abstract

**Purpose:**

To evaluate structural grading and quantitative segmentation of foveal hypoplasia using handheld OCT, versus preferential looking (PL), as predictors of future vision in preverbal children with infantile nystagmus.

**Design:**

Longitudinal cohort study.

**Participants:**

Forty-two patients with infantile nystagmus (19 with albinism, 17 with idiopathic infantile nystagmus, and 6 with achromatopsia) were examined.

**Methods:**

Spectral-domain handheld OCT was performed in preverbal children up to 36 months of age. Foveal tomograms were graded using our 6-point grading system for foveal hypoplasia and were segmented for quantitative analysis: photoreceptor length, outer segment (OS) length, and foveal developmental index (FDI; a ratio of inner layers versus total foveal thickness). Patients were followed up until they could perform chart visual acuity (VA) testing. Data were analyzed using linear mixed regression models. Visual acuity predicted by foveal grading was compared with prediction by PL, the current gold standard for visual assessment in infants and young children.

**Main Outcome Measures:**

Grade of foveal hypoplasia, quantitative parameters (photoreceptor length, OS length, FDI), and PL VA were obtained in preverbal children for comparison with future chart VA outcomes.

**Results:**

We imaged 81 eyes from 42 patients with infantile nystagmus of mean age 19.8 months (range, 0.9–33.4 months; standard deviation [SD], 9.4 months) at the first handheld OCT scan. Mean follow-up was 44.1 months (range, 18.4–63.2 months; SD, 12.0 months). Structural grading was the strongest predictor of future VA (grading: *r* = 0.80, *F* = 67.49, *P* < 0.0001) compared with quantitative measures (FDI: *r* = 0.74, *F* = 28.81, *P* < 0.001; OS length: *r* = 0.65; *F* = 7.94, *P* < 0.008; photoreceptor length: *r* = 0.65; *F* = 7.94, *P* < 0.008). Preferential looking was inferior to VA prediction by foveal grading (PL: *r* = 0.42, *F* = 3.12, *P* < 0.03).

**Conclusions:**

Handheld OCT can predict future VA in infantile nystagmus. Structural grading is a better predictor of future VA than quantitative segmentation and PL testing. Predicting future vision may avert parental anxiety and may optimize childhood development.

Nystagmus is a condition of constant, involuntary to-and-fro movements of the eyes occurring in 24 per 10 000 people.^1^ Onset is usually in early infancy, which can be alarming for parents and families, especially because preverbal children are unable to communicate their level of vision. Parents initially may believe the child is blind or has severely impaired vision, and their reaction can include fear, anxiety, and uncertainty over their child’s future.^2^ Because great variation exists in visual prognosis, predicting future vision could avert anxiety and help parents plan adjustments to support the child, particularly to optimize development and educational attainment. Targeted interventions may include accessible learning materials and toys, as well as support with safe mobility and daily living skills.^2^

OCT is a noninvasive imaging technique that provides ultra–high-resolution cross-sectional scans of the retina and optic nerve within seconds. Recently, a handheld spectral-domain OCT has been developed for clinical use in the pediatric population. It has overcome the limitations of the conventional adult OCT system and is demonstrably reliable, including in nystagmus.^3^, ^4^, ^5^ Normal retinal^6^ and optic nerve^7^ development recently were described in vivo using handheld OCT. Diseases investigated with handheld OCT include retinopathy of prematurity and other retinal disorders, nystagmus, trauma, optic nerve disease, intraocular tumors, and central nervous diseases.^3^^,^^5^^,^^8^

Normal development of the human fovea has been traced from 22 weeks’ gestation to 45 months postpartum using ex vivo histologic specimens.^9^ This is characterized by 3 developmental processes occurring at the fovea: (1) centrifugal displacement of inner retinal layers, (2) cone photoreceptor specialization, and (3) centripetal migration of cone photoreceptors.^9^ These stages are seen on OCT as the following morphologic features of the fovea, respectively: (1) formation and deepening of foveal pit with outward displacement, termed *extrusion*, of the plexiform layers; (2) outer segment (OS) lengthening; and (3) outer nuclear layer (ONL) widening.^10^ Failure of any of these processes results in foveal underdevelopment, called *foveal hypoplasia*, causing reduced visual acuity (VA). Foveal hypoplasia often is associated with infantile nystagmus.^10^ Infantile nystagmus is associated with conditions including albinism and aniridia (mutations of the *PAX6* gene) or may be idiopathic. So-called typical foveal hypoplasia is characterized by an underdeveloped fovea wherein the lining of individual retinal layers is intact.^10^ Early onset degeneration of the outer retina, as in achromatopsia, also causes abnormal persisting inner retinal layers, but because the underlying photoreceptors are disrupted, this has been termed *atypical foveal hypoplasia*.^10^^,^^11^

Structural grading of OCT morphologic features can enable classification of foveal hypoplasia based on severity. Thomas et al^10^ proposed a structural grading scheme for foveal hypoplasia based on OCT data from adults and older children that has become a widely used scheme, with grades 1 to 4 representing the most to least developed fovea, whereas the atypical grade represents disruption of the photoreceptors seen in achromatopsia. Each of the grades corresponds to statistically distinct VA levels. A key recommendation is validation of the scheme using an independent dataset. More recently, Wilk et al^12^ identified 2 subsets of grade 1 hypoplasia and proposed to subdivide these into grade 1a, in which nearly normal pit metrics (depth, diameter, volume within 2 standard deviations [SDs] of the normal average, or a combination thereof) are observed, and grade 1b, in which the pit is only a shallow indent (see “Methods”: Fig 1 for structural grading scheme used by the present study and Figure 2 for accompanying grading algorithm).

**Figure 1:**
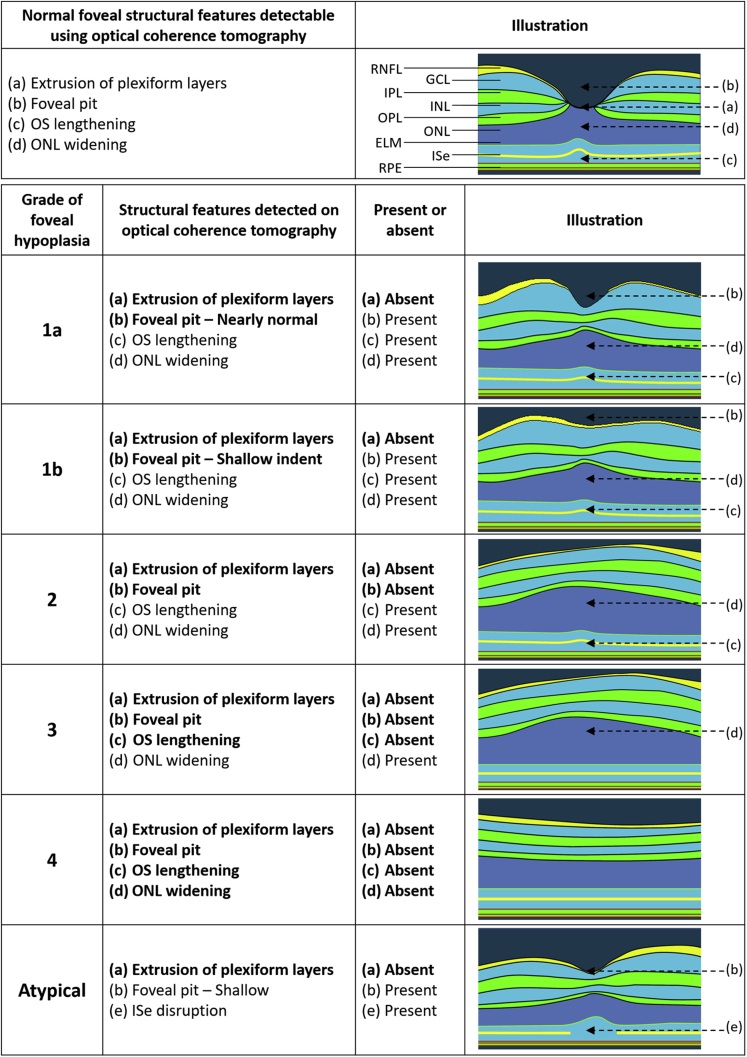
Schematic demonstrating the Leicester Grading System for Foveal Hypoplasia, showing features of a normal fovea detectable using OCT, followed by the features of typical and atypical grades of foveal hypoplasia. The normal fovea features outward displacement, termed *extrusion*, of the plexiform layers. All grades of foveal hypoplasia feature continuation of the plexiform layers. Grade 1a foveal hypoplasia is associated with a nearly normal pit resembling a “V” shape and outer segment (OS) lengthening, and outer nuclear layer (ONL) widening relative to the parafoveal OS and ONL lengths, respectively. Grade 1b foveal hypoplasia is associated with a shallow indent and OS lengthening and ONL widening relative to the parafoveal OS and ONL lengths, respectively. In grade 2 foveal hypoplasia, all features of grade 1 are present except that no pit is present. Grade 3 foveal hypoplasia represents all features of grade 2, except no lengthening of the OS segment is present. Grade 4 foveal hypoplasia represents grade 3, except no ONL widening at the fovea (termed *fovea plana*) is present. Atypical foveal hypoplasia is characterized by a shallow foveal pit and disruption of the inner segment ellipsoid (ISe) band forming a hyporeflective zone. ELM = external limiting membrane; GCL = ganglion cell layer; ILM = internal limiting membrane; INL = inner nuclear layer; IPL = inner plexiform layer; OPL = outer plexiform layer; RNFL = retinal nerve fiber layer; RPE = retinal pigment epithelium. Reprinted and adapted with permission from Thomas MG, Kumar A, Mohammad S, et al. Structural grading of foveal hypoplasia. *Ophthalmology*. 2011;118(8):1653–1660. Copyright © 2011 Elsevier.

**Figure 2:**
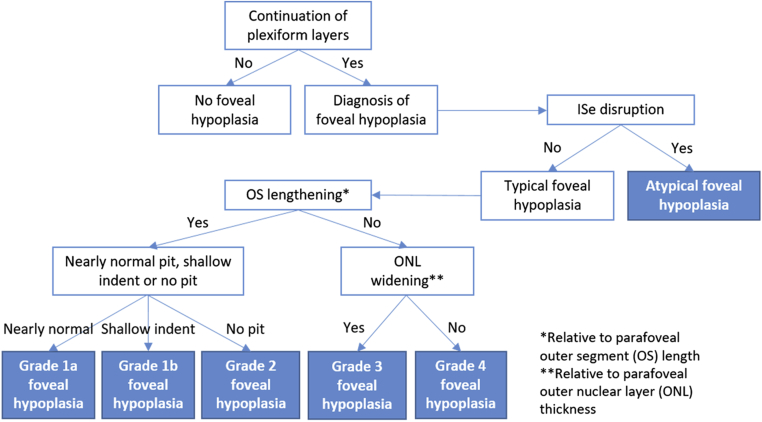
Algorithm showing the Leicester Grading Scheme for Foveal Hypoplasia. ISe = inner segment ellipsoid; ONL = outer nuclear layer; OS = outer segment.

An alternative method of evaluating foveal hypoplasia is quantitative segmentation. This involves measuring the thickness of different foveal layers. Mohammad et al^13^ demonstrated that total photoreceptor thickness and OS length are correlated highly with VA in adults with albinism. The ratio of inner layer versus total foveal thickness, termed the *foveal developmental index* (FDI), correlate with VA in albinism.^14^

The gold standard test for VA assessment in older children is logarithm of the minimum angle of resolution (logMAR) chart VA testing, beginning at 4 years of age.^15^ From 0 to 3 years of age, the gold standard test to assess VA is preferential looking (PL), based on the principle that young children tend to fixate to a pattern stimulus in preference to a plain field.^16^ However, it can be difficult to assess young children with nystagmus using PL because of their constant eye movements^17^; hence, it would be of great value to identify a better-tolerated and easier office-based method for VA prediction, such as using handheld OCT. Visual evoked potentials may represent another option for estimating VA in infantile nystagmus,^18^ but this is time consuming and difficult to perform, requiring specialist diagnostic services.

In this longitudinal cohort study, we harnessed the potential of handheld OCT for the diagnosis and prognosis of foveal hypoplasia in young children. The primary aim was to validate a structural grading system for foveal hypoplasia in infants and young children with infantile nystagmus to predict future vision. Secondary aims were (1) to assess whether structural grading is comparable with quantitative segmentation in predicting future vision, (2) to compare the predictive power of the grading system with PL VA results obtained on first examination, and (3) to determine whether the grade remains stable over time.

## Methods

### Study Design and Participants

This was a prospective, longitudinal cohort study of patients with infantile nystagmus recruited in a quaternary referral center in Leicester, United Kingdom. Longitudinal data were collected between February 20, 2012, and January 30, 2018. This study is reported according to the Strengthening the Reporting of Observational Studies in Epidemiology statement. The study adhered to the tenets of the Declaration of Helsinki, and ethics committee approval was granted by the East Midlands—Nottingham 2 Research Ethics Committee. Consent was obtained from all parents or guardians of study participants.

Inclusion criteria were as follows: (1) diagnosis of infantile nystagmus, (2) age of 36 months or younger at first handheld OCT scan, and (3) age of at least 42 months or older and ability to participate in chart logMAR visual acuity test on follow-up visit. Exclusion criteria included unsuccessful acquisition of handheld OCT scan at first visit, loss to follow-up, scans of inadequate quality for structural grading, and coexisting neurologic problems. If the handheld OCT scan was successful in only 1 eye, then the other eye was excluded from analysis. If the handheld OCT scan was unsuccessful in both eyes, then the participant was excluded from analysis. Examination 1 involved handheld OCT and full ophthalmic examination including refraction. Examination 2 involved full ophthalmic examination and best-corrected VA measurement when the participant was old enough to participate in chart logMAR vision testing. This age group was deemed clinically important because it approaches or coincides with primary school education. In addition, longitudinal handheld OCT scans were obtained.

Diagnosis of albinism was made based on abnormal lateralization on visual evoked potentials, iris transillumination defects, skin hypopigmentation, and if clinical uncertainty existed, genetic testing.^19^^,^^20^ Diagnosis of achromatopsia was made based on typical features, including light sensitivity, small amplitude nystagmus, inner segment ellipsoid disruption identified on OCT, as well as confirmatory electrodiagnostic and genetic testing.^10^ Idiopathic infantile nystagmus was diagnosed in children with nystagmus and normal ophthalmic examination results; normal visual evoked potential results; and electroretinography results, confirmation on genetic testing (i.e., pathogenic *FRMD7* mutation), or both.

### Handheld OCT Image Acquisition, Grading, and Segmentation

A noncontact, spectral-domain, handheld OCT scanner (ENVISU C class 2300 [Leica Microsystems, Wetzlar, Germany]; <4-μm axial resolution) was used to image the foveae of all participants in an outpatient setting without sedation. Visual fixation devices were used to minimize movement during imaging; these included toys, books, and cartoons. The acquisition protocol used a 10×10-mm scanning window. The 3-dimensional raster scan program for both scan sequences comprised 100 B-scans and 500 A-scans per B-scan line. The acquisition time was short (1.9 seconds) to facilitate successful image acquisition with minimal disruption of quality, thereby avoiding measurement bias.

The handheld OCT imaging protocol involved scanning the right eye followed by the left eye. The en face view was used to identify the optic nerve as a landmark for identification of the fovea. The region temporal to the optic nerve was navigated frame by frame until the most central foveal B-scan was found. Where a foveal pit was present, the B-scan with the deepest pit was selected for grading and segmentation. Where no pit was present, the B-scan with the highest OS peak or, if absent, that with the highest ONL dome was selected. If no foveal landmarks were present (termed *fovea plana*), a B-scan was selected within the central foveal area. We repeated scans 3 times per eye, selecting the highest quality scan in which the retinal layers were discernible most easily for analysis. If unsuccessful, scans were repeated until an adequate quality image was achieved or until the child ceased to participate. The total examination time typically took up to 10 minutes per child. All OCT scans were graded by 2 graders (S.R.R. and M.G.T.) to assess intergrader reliability. All OCT scans from examination 1 were segmented for quantitative analysis. Segmentation was performed using ImageJ software version 1.48 (National Institutes of Health, Bethesda, MD).

**Figure 3:**
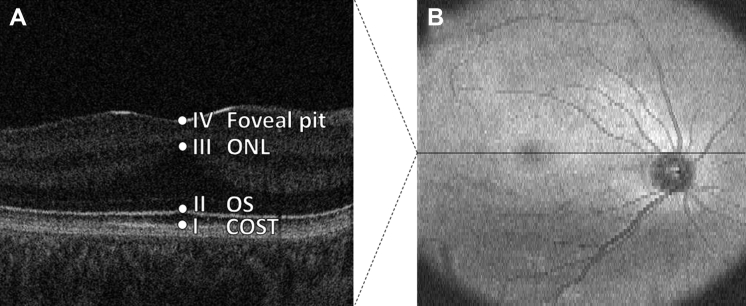
Images showing parameters for quantitative segmentation analysis: (**A**) central foveal B-scan and (**B**) en face fundal view. Three metrics were included in the segmentation analysis: (1) outer segment (OS) length, that is, the distance between point I (cone outer segment tips [COST]) and point II (OS); (2) photoreceptor length, that is, the distance between point I and point III (outer nuclear layer [ONL]); and (3) foveal developmental index, that is, the ratio of the distance between point I and point III divided by the distance between point I and point IV (foveal pit).

Structural grading was based on the scheme described by Thomas et al,^10^ with grade 1 subdivided into 1a and 1b as per Wilk et al.^12^ The grading scheme is displayed in Figure 1; we refer to this as the Leicester Grading System for Foveal Hypoplasia. The accompanying grading algorithm is displayed in Figure 2. Parameters for segmentation are illustrated in Figure 3.

### Visual Acuity Measurement

During examination 1, PL VA testing using Keeler Acuity Cards (Keeler Ltd, Windsor, United Kingdom) was performed where possible; cards were held vertically to determine more easily responses in horizontal nystagmus, thereby avoiding measurement bias.^17^ During examination 2, the Keeler LogMAR Crowded Acuity Test, which uses crowded letter optotypes on charts, was used as the gold standard VA test provided the child could cooperate.^21^ For children who could not cooperate, the Crowded Kay LogMAR Picture Test was used.^21^ This test involves matching easily recognizable pictures (house, car, star, apple, boot, and duck) and is easier for children who cannot match letters.^21^ Where possible, cycloplegic refraction was performed before examinations 1 and 2. Mean spherical equivalent for this cohort was +2.38 (range, –8.75 to 8.38; SD, ±3.22).

### Statistical Analysis

Our power calculation demonstrated that 38 patients were required to achieve power (α = 0.05; β = 0.1; *r* = 0.5), based on the null hypothesis that the correlation is 0. Data were analyzed using linear mixed-model regression analysis, including grade of foveal hypoplasia and eye recorded (right or left) in the model; the dependent variable was chart-tested logMAR VA recorded at examination 2. Independent variables recorded at examination 1 included grade of foveal hypoplasia, quantitative segmentation parameters (OS length, ONL length, and FDI), and PL VA in logMAR. Age in months also was included in the model for PL testing because the VA result is expected to improve with age.^16^ These analyses were computed using SPSS Statistics version 22.0. (IBM Corp., Armonk, NY). Agreement between 2 graders was reported to assess the validity of the grading system. Agreement of grades over time was reported to explore whether grades remained stable over time.^22^

## Results

### Baseline Characteristics

Of 47 patients enrolled in this study, data from 42 (89%) were included: 2 patients were lost to follow-up, and 3 patients did not cooperate with handheld OCT scanning. During examination 1, we obtained OCT scans of 81 eyes from 42 patients with a mean age 19.8 months (range, 0.9–33.4 months; SD, 9.4 months); 3 right-eye tomograms of 3 patients were of inadequate quality for grading and thus were excluded. Adequate power therefore was achieved (n = 38; α =0.05; β = 0.1; *r* = 0.5). Mean follow-up after examination 2 was 44.1 months (range, 18.4–63.2 months; SD, 12.0 months). Sixteen patients were female, and 26 were male. During examination 2, logMAR VA using letter optotypes (Crowded Keeler’s testing) was obtained successfully in 39 patients (93%), with all children being optimally refracted; 3 patients could not read letter optotypes and hence were tested with Kay’s LogMAR Picture Cards. Baseline characteristics of patients are listed in Table 1. The cohort included children with albinism (n = 19 patients), idiopathic infantile nystagmus (n = 17), and achromatopsia (n = 6).

**Table 1:** Baseline Characteristics of Cohort

Variable	Data
No. of eyes (patients)	81 (42)
Albinism	38 (19)
IIN	31 (17)
Achromatopsia	12 (6)
Gender, no.	
Male	26
Female	16
Age at examination 1 (mos)	
Mean (range)	19.8 (0.9–33.4)
SD	9.4
Age at examination 2 (mos)	
Mean (range)	63.9 (44.7–88.8)
SD	11.6
Follow-up (mos)	
Mean (range)	44.1 (18.4–63.2)
SD	12.0

IIN = idiopathic infantile nystagmus; SD = standard deviation.

### Grading of Foveal Hypoplasia

**Figure 4:**
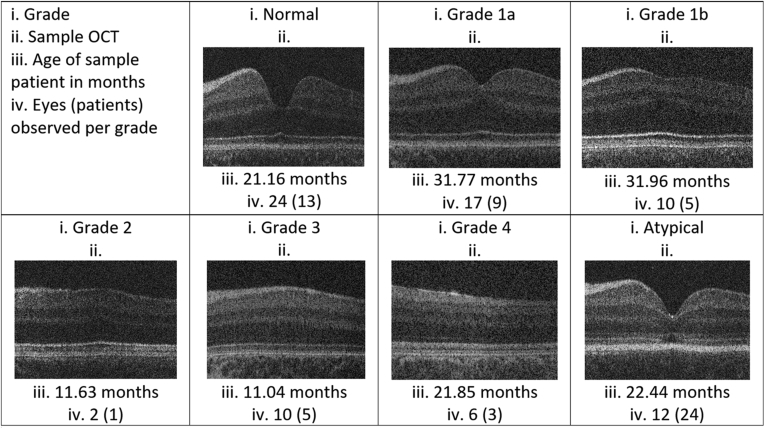
Examples of OCT scans for each grade of foveal hypoplasia.

**Figure 5:**
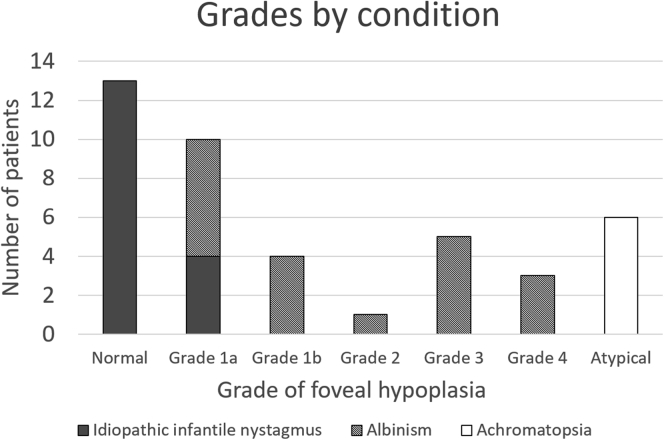
Bar graph showing the breakdown of grades by condition.

Using the grading scheme alone, the intergrader correlation coefficient was 0.96 when subdividing grade 1 into 1a and 1b, suggesting that the infant grading system is robust. There was disagreement for 3 eyes from 3 patients during the masked grading by the second grader. Each case of disagreement was over what constituted a nearly normal pit as per grade 1a versus a shallow indent as per grade 1b; however, total agreement ensued after discussion and training, which involved studying the treatment algorithm and discussing examples of 1a versus 1b pits. Agreement between graders 1 and 2 before training is displayed in the Table S1 (available at www.aaojournal.org). Foveal hypoplasia was identified in 57 eyes according to our structural grading scheme, which included grade 1a (n = 17), grade 1b (n = 10), grade 2 (n = 2), grade 3 (n = 10), grade 4 (n = 6), and atypical foveal hypoplasia (n = 12). Normal tomograms were seen in 24 eyes. Figure 4 displays samples of handheld OCT central B-scans per grade. Figure 5 displays a breakdown of diagnoses per grade.

### Prediction of Future Visual Acuity

**Figure 6:**
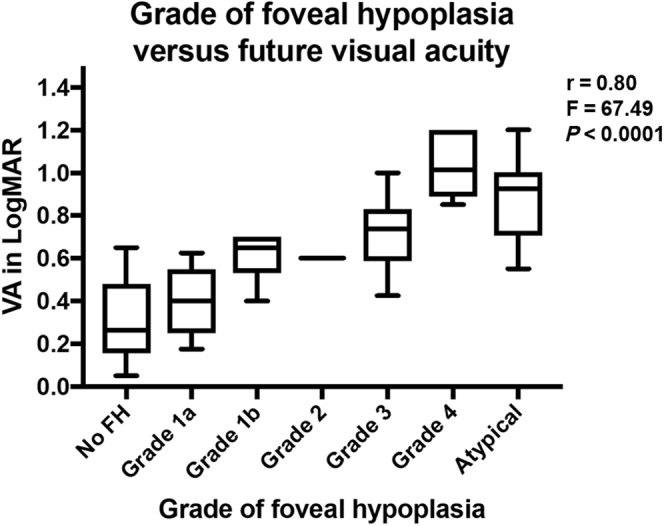
Box-and-whisker plot showing the typical grade of foveal hypoplasia during examination 1 versus visual acuity (VA) during examination 2. FH = foveal hypoplasia; logMAR = logarithm of the minimum angle of resolution.

Figure 6 is a box-and-whisker plot displaying the grade of foveal hypoplasia during examination 1 versus VA during examination 2. Median and interquartile range (IQR) per grade were as follows: no foveal hypoplasia: median, 0.26; IQR, 0.27; grade 1a: median, 0.41; IQR, 0.32; grade 1b: median, 0.65; IQR, 0.16; grade 2: median, 0.60; IQR, 0.00; grade 3: median, 0.74; IQR, 0.22; grade 4: median, 1.01; IQR, 0.23; atypical: median, 0.93; IQR, 0.28. Handheld OCT scans of gradable quality were obtained successfully from 81 of 90 eyes (90.0%). The Leicester Grading System for Foveal Hypoplasia demonstrated strong predictive power for future VA (*r* = 0.80, *F* = 67.49, *P* < 0.0001). This grading system correctly predicted future VA within 0.3 logMAR in 78 of 81 eyes (96.3%) and within 0.2 logMAR in 60 of 81 eyes (74.1%).

### Quantitative Segmentation Analysis

All quantitative segmentation parameters demonstrated statistically significant associations with future VA in typical foveal hypoplasia but none as strongly as structural grading (OS length: *r* = 0.65; *F* = 7.94; *P* = 0.008; photoreceptor length: *r* = 0.65; *F* = 6.90; *P* = 0.01; FDI: *r* = 0.74; *F* = 28.81; *P* < 0.001). Scatterplots showing quantitative segmentation analyses for examination 1 OCT scans versus VA during examination 2 are shown in Figure S1 (available at www.aaojournal.org).

### Preferential Looking

Preferential looking testing during examination 1 was assessed successfully in 49 of 72 eyes (68.1%). Preferential looking was a poorer predictor of future VA (*r* = 0.42; *F* = 3.12; *P* = 0.03) compared with handheld OCT. Age did not have a statistically significant effect (*P* = 0.09). Preferential looking testing correctly predicted future VA within 0.3 logMAR in 19 of 49 eyes (38.8%) and within 0.2 logMAR in 17 of 49 eyes (34.7%).

### Longitudinal Grading

Longitudinal OCT scans were obtained in 80 eyes from 40 patients with mean follow-up 40.5 months (range, 10.4–62.3 months; SD, 15.7 months). The longitudinal intragrader correlation coefficient was 1.0, because all grades remained the same over time.

## Discussion

We have conducted a longitudinal study using handheld OCT to predict future vision in infants and young children with nystagmus. In foveal hypoplasia, the grading scheme^10^^,^^12^ has been validated and demonstrated the strongest prediction of vision compared with quantitative measures and PL in this cohort. The intergrader reliability testing demonstrated that this scheme is robust. All grades remained stable throughout the study period. In infants and young children, all typical grades achieved VA approximately 2 lines worse than that predicted by grading in adults and older children,^10^ in whom VA per grade was as follows: grade 1: median, 0.20; IQR, 0.12; grade 2: median, 0.44; IQR, 0.18; grade 3: median, 0.60; IQR, 0.0; grade 4: median, 0.78; IQR, 0.11; atypical: median, 1.0; IQR, 0.08.^10^ In infants and young children, they were as follows: no foveal hypoplasia, 0.26; grade 1, 0.41; grade 2, 0.60; grade 3, 0.74; grade 4, 1.01; and atypical, 0.93. Because only 1 patient with grade 2 foveal hypoplasia was recruited in this study, results for grade 2 cannot be generalized. These differences could be the result of VA still undergoing development and cooperation or ability for VA testing improving with increasing age.^23^ The slightly better predicted VA in young children with atypical foveal hypoplasia in our study compared with that in the study in adults and older children likely is because achromatopsia may be a progressive disease.^24^

### Grading versus Quantitative Segmentation

Our study revealed that grading was a stronger predictor of vision compared with quantitative segmentation. An explanation could be that the grading system incorporates all key elements of foveal development, whereas measurement of FDI, OS length, and photoreceptor length represents only individual developmental landmarks. However, quantitative segmentation may give insight into subtle differences within a single grade, which may be particularly useful in achromatopsia, represented solely in 1 atypical grade. Longitudinal quantitative measurements also may help in the monitoring of patients undergoing emerging therapies. For example, measurements of the ONL in achromatopsia, which changes with increasing age,^24^ are particularly important to determine the degree of cone degeneration in view of gene therapy.

Grading can be performed rapidly in the clinic, whereas quantitative segmentation requires special training and takes approximately 20 minutes per patient. Furthermore, grading can help clinicians make appropriate decisions regarding management and investigation. For example, if VA is poorer than expected according to grading, there should be raised suspicion of other pathologic features limiting the VA. This may include other retinal pathologic features such as retinal dystrophy, cortical or neurologic disorders, amblyopia, conditions affecting the anterior segment, or suboptimally corrected refractive errors. Similarly, if a patient has poor vision consistent with a high degree of foveal hypoplasia, it is likely that the limiting factor for poor VA is accounted at the retinal level.

### Grading versus Preferential Looking

Preferential looking was a poor predictor of future VA, tending to underestimate visual potential in nystagmus. Constant eye movement may increase difficulties of assessing whether the child has correctly identified test cards.^17^ Suboptimal refraction during examination 1 may have contributed to the poor correlation between PL and chart VA. Mean chart VA predicted by PL in our cohort was 1.0 logMAR, better than a previously published figure in another young cohort with infantile nystagmus with mean age of 43 months (1.7 logMAR), but still poor.^25^ Moreover, the success rate in PL was poor (68.1%) compared with handheld OCT (90.0%). Dubowitz et al^26^ achieved a similar success rate of 70% (n = 96) for PL in children with retinopathy of prematurity, also associated with foveal hypoplasia, of a similar age to those in our study. In our study, PL testing correctly predicted future VA within 3 lines in only 38.8% of children compared with 96.3% of children for handheld OCT.

### Can the Fovea Be Graded from Birth?

Our grading system involves identifying the presence or absence of intact inner segment ellipsoid, foveal pit, OS lengthening, and ONL widening. Histologic and OCT studies have demonstrated that all of these landmarks can be identified from birth except OS elongation, which continues postnatally for up to 5 months.^6^^,^^27^ Hence, it is possible to determine all grades apart from grades 2 and 3 from birth, because an infant may be assigned grade 3 inappropriately before OS elongation has occurred. In this study, no patient younger than 5 months with grade 2 or 3 foveal hypoplasia was identified. However, we successfully obtained scans from patients who were 1 month and 3 months of age with grade 4 and atypical foveal hypoplasia, respectively. Longitudinal OCT scans revealed that all grades remained the same until the end of the study period; thus, adding age into the regression model would not impact the correlation between grading and future VA.

### Study Strengths and Limitations

This longitudinal cohort study has used handheld OCT to predict future vision in infantile nystagmus. This helps to reassure and enable patients and families to optimize the development and educational attainment of the child during this crucial age, as well as helping clinicians in their decision-making process for investigation and management. A powered cohort of infants and young children has been achieved with adequate follow-up until the child can participate with chart VA, approaching or coinciding with commencement of primary school education. Our grading system demonstrated an intergrader coefficient of 0.96, suggesting that it is robust. Our grading system showed lower prediction of VA than previously found in older patients.^10^ This is likely because of less cooperation with logMAR VA testing and an immature visual system.^23^ Nystagmus characteristics and head posture may influence VA; however, even without taking these factors into account, we found very good correlation between foveal structure and later VA. In the future, a multivariate model including foveal structure and nystagmus could be calculated to investigate whether VA prediction can be refined.

A limitation of our study is that we are currently able to predict VA only in childhood, rather than adulthood, because of the recent availability of handheld OCT. However, it is still of great value to predict an infant’s VA at the time the child commences primary education. It is likely that VA will continue to improve based on adult data.^10^ Another limitation is that only 3 causes of foveal hypoplasia were represented within this cohort; the grading system was not applied to other conditions associated with foveal hypoplasia such as *PAX-6* mutation,^28^ retinopathy of prematurity,^29^ nanophthalmos,^30^ and other photoreceptor dystrophies. Only 2 eyes from 1 patient fulfilled the criteria for grade 2; therefore, median VA predicted from this single patient (0.60) cannot be generalized. With respect to image acquisition, if 3 scans per eye were unsuccessful, patients could not be included. However, most eyes (90%) were scanned successfully.
