# Supplementary material for: Can Structural Grading of Foveal Hypoplasia Predict Future Vision in Infantile Nystagmus? A Longitudinal Study
Source: Ophthalmology. 2020 Apr;127(4):492–500. doi: 10.1016/j.ophtha.2019.10.037 (PMC7105819; doi:10.1016/j.ophtha.2019.10.037)
Supplement: Table S1 [file mmc1.pdf]

Grade	Grader 1 tally	Grader 2 tally
No foveal hypoplasia	24	24
Grade 1a	17*	14*
Grade 1b	10*	13*
Grade 2	2	2
Grade 3	10	10
Grade 4	6	6
Atypical foveal hypoplasia	12	12

Supplemental Table: Agreement between Graders 1 and 2 prior to training. *Key: * denotes disagreement over three eyes between Grades 1a and 1b, with total agreement achieved following training.*
