# Supplementary material for: Can Structural Grading of Foveal Hypoplasia Predict Future Vision in Infantile Nystagmus? A Longitudinal Study
Source: Ophthalmology. 2020 Apr;127(4):492–500. doi: 10.1016/j.ophtha.2019.10.037 (PMC7105819; doi:10.1016/j.ophtha.2019.10.037)
Supplement: Figure S1 [file mmc2.pdf]

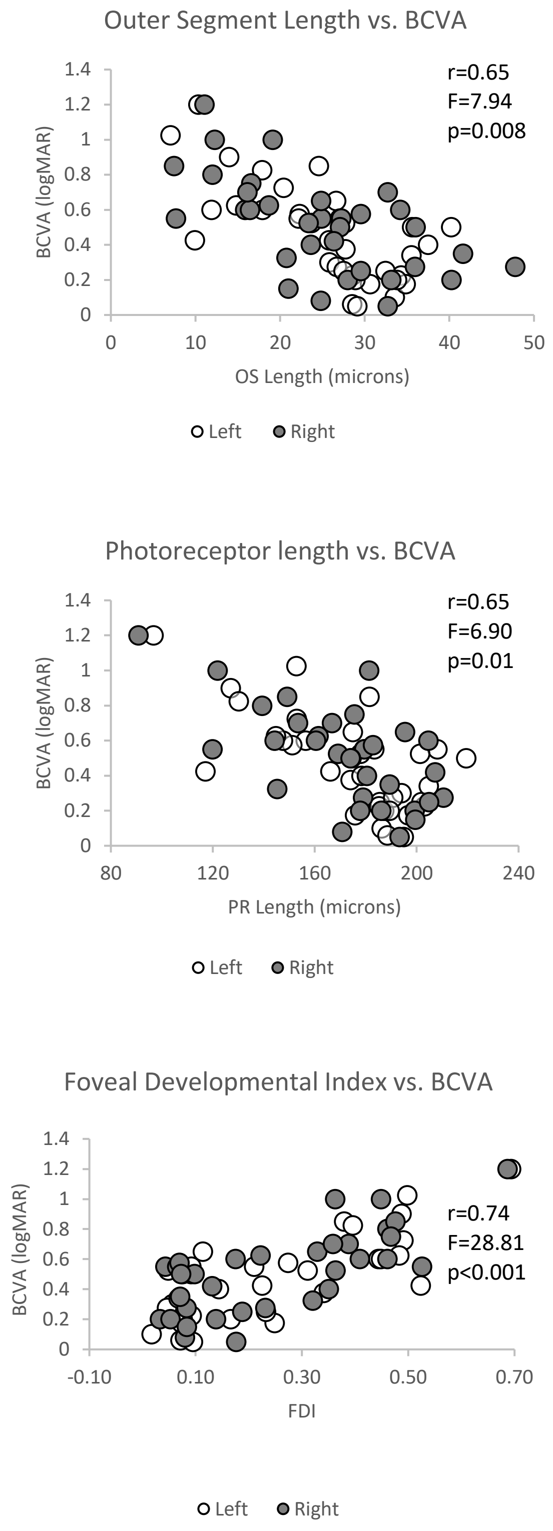

eFigure 1: Quantitative segmentation analyses versus future visual acuity in typical foveal hypoplasia. *BCVA = best corrected visual acuity.*
